# Polarization-Dependent Anisotropy of LIPSSs’ Morphology Evolution on a Single-Crystal Silicon Surface

**DOI:** 10.3390/mi15020200

**Published:** 2024-01-29

**Authors:** Mengting Liu, Baole Lu, Jing Lv, Jiang Wang, Chen Li, Guodong Zhang, Jintao Bai, Razvan Stoian, Guanghua Cheng

**Affiliations:** 1 Institute of Photonics & Photon Technology, Northwest University, Xi’an 710127, China; mengtingliu2023@163.com (M.L.); lubaole1123@163.com (B.L.); baijt@nwu.edu.cn (J.B.); 2 School of Artificial Intelligence, Optics and Electronics (iOPEN), Northwestern Polytechnical University, Xi’an 710072, China; lvj1228@nwpu.edu.cn (J.L.); wjiang@nwpu.edu.cn (J.W.); guodongzhang@nwpu.edu.cn (G.Z.); 3 College of Mechanical and Electrical Engineering, Shaanxi University of Science and Technology, Xi’an 710021, China; ichen@sust.edu.cn; 4 Laboratoire Hubert Curien, UMR 5516 CNRS, Université Jean Monnet, 42000 Saint Etienne, France; razvan.stoian@univ-st-etienne.fr

**Keywords:** LIPSSs, picosecond laser, polarization-dependent anisotropy

## Abstract

Utilizing the principle of laser-induced periodic surface structures (LIPSSs), this research delves into the morphological evolution of single-crystal silicon surfaces irradiated by a near-infrared picosecond laser through a scanning mode. With the increase in laser energy density, the nanostructure morphology on single-crystal silicon surfaces induced by incident lasers with different polarization directions sequentially produces high spatial-frequency LIPSSs (HSFLs) with a period of 220 nm ± 10 nm parallel to the laser polarization, low spatial-frequency LIPSSs (LSFLs) with a period of 770 nm ± 85 nm perpendicular to the direction of the polarization, and groove structures. Furthermore, by varying the angle between the laser polarization and the scanning direction, the study examined the combined anisotropic effects of the laser polarization scanning direction angle and the laser polarization crystal orientation angle on the genesis of LIPSSs on single-crystal silicon (100) surfaces. The experiments revealed polarization-related anisotropic characteristics in the morphology of HSFLs. It was found that when the polarization angle approached 45°, the regularity of the LSFLs deteriorated, the modification width decreased, and the periodicity increased. This is critical for the precise control of the LSFLs’ morphology.

## 1. Introduction

Laser-induced periodic surface structures (LIPSSs) are a phenomenon in which periodically distributed nanostripes are formed within the spot area when a laser irradiates the surface of a material [1]. This may be caused by the inhomogeneous deposition of photon energy due to the optical field pattern established by the interference between the incident light and the surface electromagnetic waves on the material surface during the irradiation of the laser pulse [2,3,4] and the subsequent hydrodynamic-driven morphology organization, as in Refs. [5,6,7]. LIPSSs are commonly found in a variety of metal [8], dielectric [9], and semiconductor materials [10]. They are a flexible and convenient nanostructure processing technology with no masks, no contact, and small material limitations [11,12,13], which are expected to overcome the challenges of complex fabrication processes, limited processing material, and high environmental requirements in micro-nano manufacturing processes, such as photolithography [14], focused ion beam [15], and electron beam lithography [16]. They have the capability to change the optical, mechanical, and biological properties of materials, presenting considerable prospects for development in the field of material modification. Currently, they have been studied on metal structural color [17], surface antibacterial performance enhancement [18], sensing [19], polarization conversion of light [20], friction properties [21], absorption enhancement of solar cells [22], and other applications, showing substantial potential applications in various fields, including medicine, optics, tribology, and energy technology, etc.

Due to the characteristics of ultrafast lasers with extremely high peak power and ultrashort pulse width, a novel phenomenon of sub-wavelength order periodicity in LIPSSs, known as high spatial frequency LIPSSs (HSFLs), is generated when it irradiates the surface of materials, has attracted widespread attention among researchers. However, the mechanism of its formation remains unclear. Silicon, as a representative semiconductor material, has been extensively investigated for the generation of LIPSSs on its surface by ultrafast laser irradiation [23]. A large-area HSFL_⊥_ can be fabricated on silicon by using a nanojoule femtosecond laser with a repetition rate of 80 MHz [24]. LSFLs and grooves were observed in a study exploring the pulse overlap ratio and energy density levels during picosecond laser irradiation [25]. By varying the laser power and sample scanning speed, three distinct types of nanostructures, namely LSFL_∥_, HSFL_⊥_, and LSFL_⊥_, were observed through photothermal-induced oxidation on Si-on-Pt hybrid ultrathin films [13]. The different types of nanostructures mentioned above are generated under different processing parameters. However, there are few reports on the atypical structure of HSFL_∥_ induced by a picosecond laser on single-crystal silicon surfaces in scanning mode. In addition, the coexistence of HSFLs and LSFLs on the same scan line under fixed processing parameters has hardly been found.

In practical application requirements, it is often necessary to consider adopting scanning methods for achieving large-scale nanostructure processing. By controlling the scanning direction, it becomes feasible to achieve the overall direction adjustment and shape control of the nanostructure. Consequently, studies in this domain have begun to focus on the influence of preformed nanostructures generated by the initial pulse during the scanning process on the unscanned regions [26,27]. At the same time, when using linearly polarized laser processing, the polarization angle of the laser provides another degree of freedom of directional control for surface pattern processing by adjusting the stripe orientation of the LIPSSs. Moreover, it is noteworthy that the majority of studies typically assume that the polarization state of linearly polarized light solely determines the direction of the stripes [23]. In addition, when using a laser to process crystalline materials, the results may be different due to disparities in the relevant crystal properties [28,29]. However, there is limited research on the effects of different relative angles among the scanning direction, crystal orientation, and laser polarization direction on the generation of LIPSSs in crystal materials. Han et al. [30] conducted related research on the surface of Si (111), analyzing the anisotropy of elliptically modified morphologies caused by enhanced energy absorption along the laser polarization direction and scattering of surface plasmon polaritons (referred to as the laser polarization scanning direction anisotropy effect). They also examined its impact on the line width and continuity of low spatial frequency laser-induced periodic surface structures (LSFLs), as reported in their study. Jiang et al. [31] mitigated the aforementioned laser polarization scanning direction anisotropic effect on the surface of Si (100) by consistently maintaining the polarization direction perpendicular to the scanning direction. This approach elucidated the anisotropic characteristics of the continuity of LSFLs that depend on crystal orientation (referred to as the laser polarization crystal orientation anisotropy effect). Previous studies individually investigated one of the two types of anisotropic effects. When employing Si (100) and concurrently varying the angle between laser polarization and scanning direction, the theoretical analysis suggests that the process of generating LIPSSs will be influenced by both the aforementioned laser polarization scanning direction effect and the laser polarization crystal orientation effect. However, what kind of polarization-dependent anisotropic characteristics will the actual LIPSSs exhibit?

In this report, the LIPSSs generated on the surface of single-crystal silicon (100) by near-infrared polarized picosecond laser irradiation is studied. The evolution of the morphology of nanostructures induced by incident laser beams with different polarization angles on the surface of single-crystal silicon materials with the increase in laser energy density is demonstrated. The combined effects of laser polarization scanning direction and laser polarization crystal orientation during the LIPSSs formation process have been investigated in an experiment. The study also examined the rules of polarization-dependent anisotropic characteristics in LSFLs’ morphology, regularity, modification width, and periodicity resulting from the interaction of the aforementioned two distinct effects.

## 2. Materials and Methods

**Figure 1:**
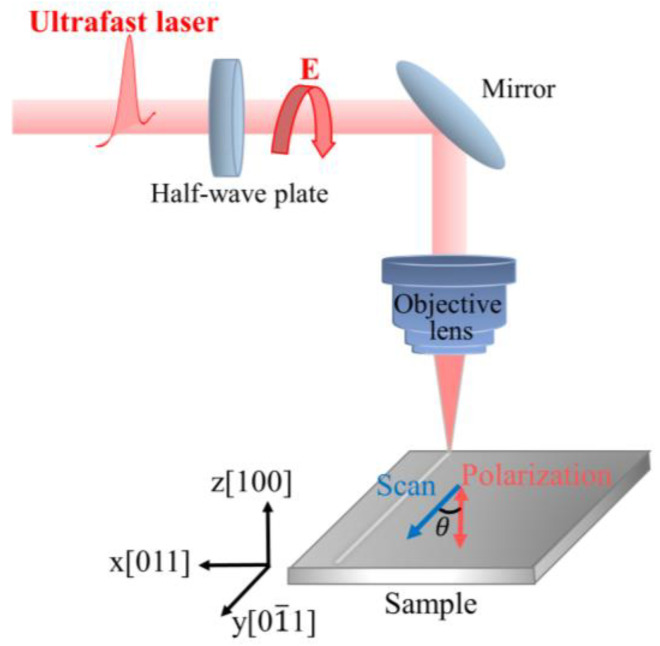
Schematic diagram of the experimental setup.

The experimental setup is shown in Figure 1. In our experiments, the sample is a single-sided polished p-type single-crystal silicon with a resistivity ranging from 0–20 Ω/cm and a crystal orientation of <100>. A Gaussian spatially distributed linearly polarized light with a wavelength of 1030 nm, repetition rate of 100 KHz, and pulse width of 1.2 ps is generated using an ultrafast laser system (Monaco, Coherent, Bangkok, Thailand). The continuous and precise adjustment of laser power is achieved through a combination of a half-wave plate and a polarizer. The laser beam is focused by an objective lens (Mitutoyo 2×, NA = 0.055, focal length f = 100 mm) and it is vertically incident onto the sample surface in air. A zero-order quartz half-wave plate in front of the objective lens is used to adjust the laser polarization direction. The three-dimensional motion platform (Aerotech ANT130) with a repeatability of ±75 nm enables precise movement of the sample within the laser focal plane. The crystal orientation, laser polarization direction, and sample scanning direction are defined in Figure 1, and the orientation of the sample’s scanning direction with respect to the crystal’s axis direction was determined by EBSD (electron backscatter diffraction).

In the experiment, the scanning direction and the crystal axis [011] are kept parallel to the *y*-axis. The angle between the laser polarization direction and the *y*-axis (i.e., the scanning direction and the crystal orientation [011]) is defined as the polarization angle θ. After laser irradiation, the sample was ultrasonically cleaned in ethanol for 6 min, and its surface morphology and microstructure modification were characterized using scanning electron microscopy (SEM, Tescan Clara GMH, Shanghai, China). Employing a MATLAB R2021a algorithm based on the principles of two-dimensional fast spatial Fourier transform, the SEM images are processed to obtain the periodicity of the LIPSS structure.

## 3. Results and Discussion

### 3.1. Morphology Evolution with Energy Density under Laser Irradiation at Different Polarization Angles

**Figure 2:**
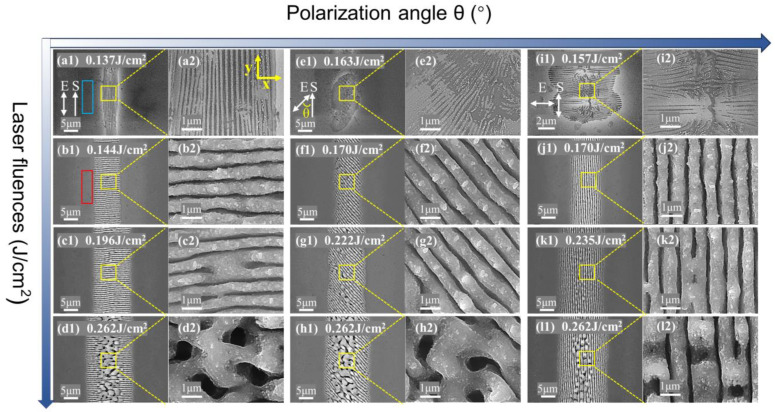
SEM images of the morphology evolution of a single-crystal silicon surface with increasing laser energy density under picosecond laser irradiation at different polarization angles. The polarization angle of (**a1**–**d1**) is 0°, and the energy densities are 0.137 J/cm^2^, 0.144 J/cm^2^, 0.196 J/cm^2^, and 0.262 J/cm^2^, respectively. (**a2**–**d2**) are the local enlarged graphs of (**a1**–**d1**), respectively. The polarization angle of (**e1**–**h1**) is 45°, and the energy densities are 0.163 J/cm^2^, 0.170 J/cm^2^, 0.222 J/cm^2^, and 0.262 J/cm^2^, respectively, and (**e2**–**h2**) are the local magnification graphs of (**e1**–**h1**), respectively. The polarization angle of (**i1**–**l1**) is 90°, the energy densities are 0.157 J/cm^2^, 0.170 J/cm^2^, 0.235 J/cm^2^, 0.262 and J/cm^2^, respectively, and (**i2**–**l2**) are local magnifications of (**i1**–**l1**), respectively. The bidirectional arrow indicates the laser polarization, the unidirectional arrow indicates the scanning direction, and the angle between them is the polarization angle θ. The blue rectangle in (**a1**) shows that, within a relatively lower range of energy densities, there are virtually no nanoparticles or ablation debris around the modified area. In contrast, the red rectangle in (**b1**) demonstrates that a higher range of energy densities leads to a greater quantity of nano-particles and ablation debris around the modified area. (**a2–l2**) provide respective magnified views of the areas within the yellow rectangles in (**a1–l1**).

The morphological evolution of the single-crystal silicon surfaces with increasing energy density was investigated under the picosecond laser irradiation with different polarization angles. The repetition rate of the laser pulse with a fixed pulse duration of 1.2 ps was set at 100 KHz and a scanning speed of 8 mm/s. When the polarization angles of the incident laser were 0°, 20°, 40°, 45°, 50°, 70° and 90°, respectively, the morphology evolution of the material surface followed the same change law. Specifically, with the increase in the laser energy density, three distinct structures were sequentially generated. These structures included the high-spatial frequency LIPSSs (HSFLs) parallel to the polarization with a period Λ of 220 nm ± 10 nm, as shown in Figure 2a,e,i, the classical low spatial frequency LIPSSs (LSFLs) with Λ of 770 nm ± 85 nm perpendicular to the polarization direction, as shown in Figure 2b,f,j, and the groove structure growing from the center of LSFLs, as shown in Figure 2d,h,l.

**Figure 3:**
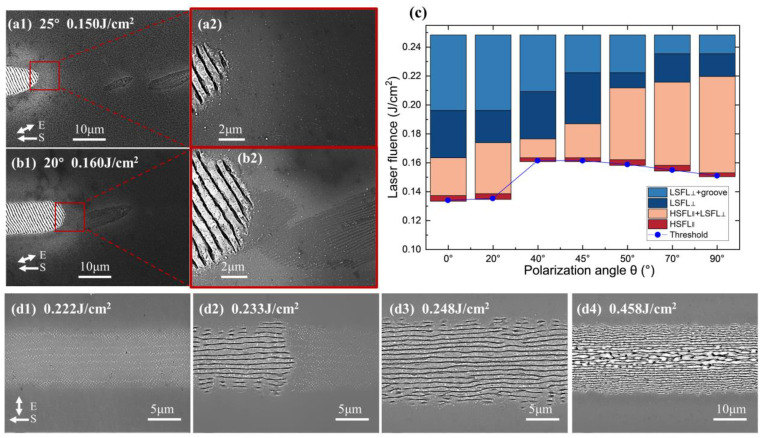
(**a**,**b**) SEM images of the mixed state of HSFL_∥_ and LSFL_⊥_ on the surface of single-crystal silicon under incident picosecond laser irradiation with a polarization angle of 25° and 20°. The corresponding energy densities of (**a**,**b**) are 0.150 J/cm^2^ and 0.160 J/cm^2^, respectively. (**a2**,**b2**) are local magnification diagrams of (**a1**,**b1**), respectively. (**c**) The energy density windows of different typical morphologies and generation threshold curves of LIPSSs generated by picosecond laser irradiation on single-crystal silicon surface at different polarization angles. (**d1**–**d4**) SEM images of the nanoparticles, mixed state of nanoparticles and LSFL_⊥_, LSFL_⊥_, and grooves on the surface of single-crystal silicon under incident femtosecond laser irradiation at a polarization angle of 90°, respectively. The corresponding energy densities of (**d1**–**d4**) are 0.222 J/cm^2^, 0.233 J/cm^2^, 0.248 J/cm^2^, and 0.458 J/cm^2^, respectively.

At the same polarization angle, in a relatively lower range of energy density (slightly lower than the multi-pulse ablation threshold), the interference of near-field scattering from small surface roughness or crystal defects with the incident laser causes inhomogeneity of the light field. And, near the material ablation threshold, the inhomogeneity energy deposition induces melting on the surface of the material, leading to the formation of HSFL_∥_ rather than ablation. Due to the Gaussian distribution of the laser spot, as the energy density increases, HSFL_∥_ initially begins to disappear at the central part of the modified region, as shown in Figure 2(i2). In the medium energy density range (equal to or slightly larger than the multi-pulse ablation threshold), the higher energy density enhances the laser ablation at the first few pulses, resulting in the generation of more and larger surface nanostructures on the material surface. This is evidenced by the appearance of nanoparticles and ablative debris in the red rectangular box in Figure 2(b1) and Figure 3a,b, which are not present in the blue rectangular box in Figure 2(a1). The generation of LSFL_⊥_ is initiated by the interference of far-field scattering generated by the larger surface roughness with the incident laser. And the interference of the excited surface plasmon waves with the incident laser and the SPP grating-assisted mechanism further enhances generation of the LSFL_⊥_. At a higher energy density (much higher than the multi-pulse ablation threshold), the initial pulse will induce greater local topographical changes. At a high repetition frequency, the overlap of the laser spot of the last pulse and the area where the previous pulse has acted forms a feedback adjustment of the morphology-driven light field distribution, resulting in a more intense and non-uniform distribution of energy deposition. And the spatial Gaussian intensity distribution of the laser beam drives the melting of the single-crystal silicon and the accumulation of strong transverse and axial inhomogeneous temperature gradients on the molten surface. It induces the destruction of the LSFL_⊥_, and then the grooves are generated through the related hydrodynamic processes, such as hot capillary force.

It is noteworthy that there is a mixed state of HSFL_∥_ and LSFL_⊥_, as shown in Figure 3a,b, that has not been discovered in previous studies. Additionally, we conducted experiments with femtosecond laser irradiation (central wavelength of 1030 nm, pulse width of 150 fs) on a single-crystal silicon (100) surface, under identical conditions to the picosecond experiments. These conditions included a constant laser scanning speed of 8 mm/s and a pulse repetition frequency of 100 kHz. As shown in Figure 3(d1–d4), with increasing laser energy density, the surface of single-crystal silicon successively formed nanoparticles, LSFL_⊥_, and grooves, without the generation of HSFL_∥_. The coexistence of observed nanoparticles and LSFL_⊥_ was noted. Moreover, in all picosecond laser irradiation experiments where HSFL_∥_ and LSFL_⊥_ coexisted, HSFL_∥_ appeared only at the leading edge of the same scan line under fixed processing parameters. This suggests that the HSFL_∥_ formed by preceding pulses altered the light field distribution of subsequent pulses and promoted energy deposition, leading to a transition from HSFL_∥_ to LSFLs. Similarly, in femtosecond laser pulse experiments, nanoparticles always appeared at the leading edge of the same scan line as LSFLs. This insight is beneficial for further understanding the formation mechanisms of LIPSSs at energy densities close to the ablation threshold. The reason why picosecond lasers generate HSFL_∥_ instead of nanoparticles, compared to femtosecond sources, could be due to the provision of suitable heat and electric fields. Experimentally, this demonstrates that the formation of HSFL_∥_ under picosecond laser irradiation and nanoparticles under femtosecond laser irradiation both promote the formation of classical LSFLs.

The energy density windows corresponding to different typical morphologies are shown in Figure 3c. In the polarization angle range of 0° to 90°, the threshold of LIPSS generation is larger as it is closer to 45°, and the threshold at 0° is smaller than 90°. This may be related to the dependence of nonlinear ionization on crystal orientation, which will cause different polarization angles to produce differences in the dielectric constant due to the difference in free electron density. In our experiment, as the angle between the laser polarization direction and the crystal axis [011] or [01$\bar{1}$] tends to be 45°, a decrease in the degree of nonlinear ionization results in an increase in the threshold.

### 3.2. Polarization-Dependent Morphology Anisotropy of LIPSSs

**Figure 4:**
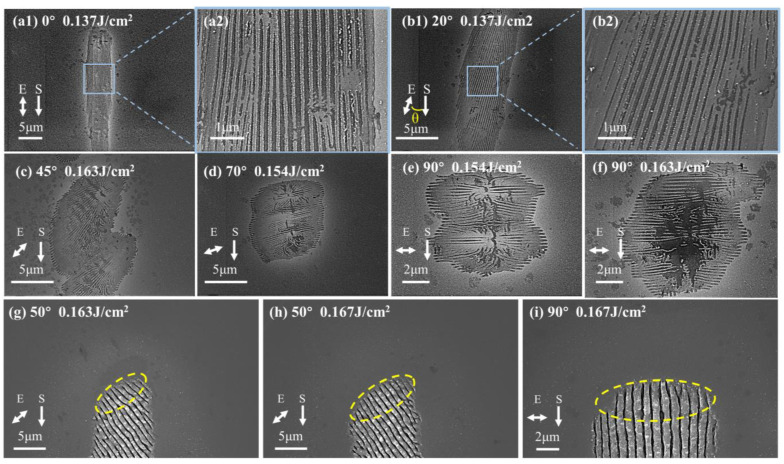
SEM images of (**a**–**f**) HSFL_∥_ and (**g**–**i**) the front segment of the LSFL_⊥_ on the surface of single-crystal silicon under irradiation at different polarization angles. The polarization angles of (**a1**), (**b1**), and (**c**–**i**) are 0°, 20°, 45°, 70°, 90°, 90°, 50°, 50°, and 90°, respectively, and the energy densities are 0.137 J/cm^2^, 0.137 J/cm^2^, 0.163 J/cm^2^, 0.154 J/cm^2^, 0.154 J/cm^2^, 0.163 J/cm^2^, 0.163 J/cm^2^, 0.167 J/cm^2^ and 0.167 J/cm^2^, respectively. (**a2**) and (**b2**) are local magnifications of (**a1**) and (**b2**), respectively. The yellow dotted ellipses in the figure schematically represent the morphology of the elliptical modification areas induced by laser pulses with different polarization states.

The HSFL_∥_ shown in Figure 4a–f and the LSFL_⊥_ shown in Figure 4g–i, which generated by incident lasers with different polarization states. They both show obvious polarization-dependent anisotropy, that is, with the change in the laser polarization direction, HSFL_∥_ forms an elliptical modified region with its long axis parallel to the laser polarization direction, instead of the circular region of the Gaussian spot. Along the laser polarization direction, the ablation of the front segment of the LSFL_⊥_ is also enhanced, which may be attributed to the enhancement of directional scattering field due to the higher energy absorption efficiency in this direction. And along the scanning direction, the modified region is connected in different overlapping ways and areas, from 0° to 90°, following a gradual change from connecting the long axis of the elliptical modified region to the minor axis.

In different polarization states, the edges of the HSFL_∥_ modified region are regular periodic fringes parallel to the laser polarization direction, as shown in Figure 4a–f. However, the regularity of the central part deteriorates, which may be related to the weakening of the feedback assistance effect of the surface stripes generated after the previous pulse on the unscanned area in the scanning direction. That is, when the direction of the fringes is the same as the scanning direction (θ = 0°), the near-field enhancement generated by the formed nanofringes has the strongest effect on the orderly guidance of subsequent fringes, and gradually decreases with the increase in the angle between them.

**Figure 5:**
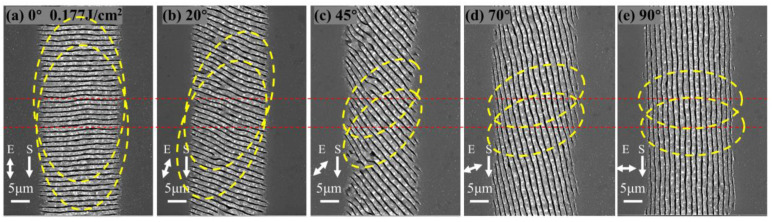
SEM images of LSFL_⊥_ on the surface of a single-crystal silicon irradiated with different polarization angles. (**a**–**e**) The polarization angles are 0°, 20°, 45°, 70°, and 90°, respectively, the energy density is 0.177 J/cm^2^, the scanning speed is 8 mm/s, and the pulse repetition rate is 100 KHz. The yellow elliptical dotted lines represent the elliptical modified regions formed when the laser irradiates the silicon surface with different polarization states and the long axis of these ellipses is parallel to the direction of laser polarization. The relative position of the modification area movement, determined by the fixed scan speed and pulse repetition rate, is indicated by a red dotted line.

LSFL_⊥_ exhibits polarization-dependent regularity anisotropy, as shown in Figure 5. Considering the enhancement of the scattered field under polarization guidance, when the polarization direction of the laser is parallel to the scanning direction (θ = 0°), the SPP excitation in the unaffected region and the coupling between the incident laser and the surface plasma wave are the strongest in the scanning direction, which is the most beneficial for improving the regularity of the stripes. As the polarization angle of the laser changes from 0° to 90°, the far-field assistance effect of SPPs gradually weakens, and the regularity gradually decreases. However, in the experimental results, as the polarization angle approaches 45°, the regularity becomes worse. The reason is that SPPs plays a role in the generation mechanism of LSFLs, but it may not be the sole contributing factor. When LSFLs are generated in the scanning processing mode, the feedback effect of light field distribution and material surface modification during the cumulative effect of pulses in the scanning direction should also be fully considered. The enhanced absorption caused by the pre-existing ripple structures, especially the extremely strong near-field enhancement at the tip of the ripple, will cause directional ablation in this direction, which guides the growth of the stripes along the original trajectory. Therefore, when the scanning direction is parallel to the stripe direction (θ = 90°), the enhancement effect on the unacted area’s local field in the scanning direction is strongest, which is most favorable for improving the regularity of the stripes. As the laser polarization angle varies from 90° to 0°, the auxiliary effect of local field enhancement gradually weakens, and the regularity gradually decreases. Considering the combined influence of the aforementioned two effects and the experimental results, it is evident that both play an important role in the generation mechanism of LSFLs in the scanning mode. And, among these angles, the regularity of 0° and 90° is relatively good, which is due to the strongest controlling effect of the far-field interference of the SPP and the most influential guiding effect of the field enhancement of the generated local morphology change, respectively. The combined effect of both factors results in the lowest regularity when the angle approaches 45°.

### 3.3. Polarization-Dependent Period and Modified Width Anisotropy of LSFL_⊥_

**Figure 6:**
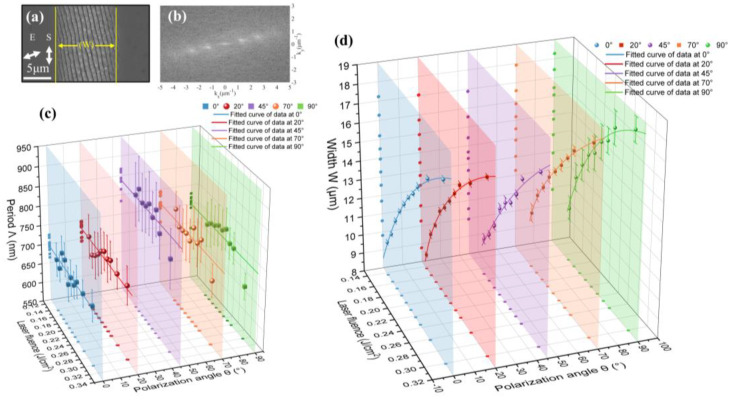
(**a**) SEM image of LSFLs generated by picosecond laser on the surface of single-crystal silicon. The polarization angle is 70°, and the energy density is 0.169 J/cm^2^. (**b**) is a fast two-dimensional space Fourier transform diagram of (**a**). Under picosecond laser irradiation at various polarization angles, (**c**) is the variation in the periodicity of the LSFLs within the laser-modified region on the single-crystal silicon surface with energy density, along with its corresponding standard deviation and the fitted curves, while (**d**) is the variation in the width of the modified region with energy density, along with its corresponding standard deviation and the fitted curves.

The characteristic parameters of LSFLs generated by laser irradiation on the surface of single-crystal silicon irradiated at different polarization angles were analyzed. The SEM image shown in Figure 6a was processed using the two-dimensional fast space Fourier transform method, as depicted in Figure 6b. Through this method, the periodicity corresponding to LSFLs, as shown in Figure 6c, was obtained. The period of LSFLs generated by irradiation under the same polarization state does not vary much with increasing energy density. The period of LSFLs generated by laser irradiation with different polarization states shows the maximum at 45° and the minimum at 0° over the entire range of energy densities. The variation in the modification width of LSFLs with increasing energy density under different polarization states is shown in Figure 6d. Under irradiation in the same polarization state, the modification width increases with increasing energy density. However, under irradiation in different polarization states, the modification width almost reaches its maximum at 90° in the entire range of energy densities. And the closer the polarization angle is to 45°, the smaller the modification width becomes, which can also be seen visually in Figure 5.

With the increase in laser energy density at the same polarization angle, the area of the modified region of LSFLs will gradually increase due to the threshold effect. And the electron density of the modified region will not increase linearly with the increase in laser energy density. Consequently, the period of LSFLs does not change much as the laser fluence increases. According to the polarization dependence of the LIPSS generation threshold in Section 3.1, at the same energy density, the closer the polarization angle of incident light is to 45°, the less energy deposited into the material, the smaller the modified area of LSFLs is, and the modified area of 0° should be greater than 90°. The reason for the minimum modification width observed at 0° in the experimental results may be that the polarization-oriented scattering field enhancement also plays an important role in the formation process of LSFLs. As revealed in Section 3.2, when the material surface is irradiated by laser beams in different polarization states, an elliptical modified region with its long axis parallel to the laser polarization direction is formed, as shown by the yellow elliptical dotted line in Figure 5. Therefore, as the laser polarization angle rotates from 0° to 90°, perpendicular to the scanning direction, the transverse width of the modified region gradually changes from the short axis length to the long axis length. On the contrary, along the scanning direction, the longitudinal length of the modified region gradually changes from the long axis length to the short axis length. In addition, at a constant scanning speed and pulse repetition frequency, the relative position shift of the modified region is depicted by the red dashed line in Figure 5. Considering the combined influence of the aforementioned factors, it is consistent with the experimental results that the transverse modified width is the largest when the polarization angle is 90° and becomes smaller as it becomes closer to 45°. The overlapping area of the modified region is minimized at the polarization angle of 45° and maximized at 0°. According to the coupling theory of SPP and the laser field assisted by LSFLs [32], the smallest period occurs at the polarization angle of 0° because of the strongest positive feedback effect, while the period is the largest at 45° where the positive feedback effect is the weakest, which is also consistent with the experimental results that the period at the polarization angle of 20° is smaller than at 70°.

### 3.4. Large-Area LIPSSs Arrays with Angled Stitching Based on Polarization Control

**Figure 7:**
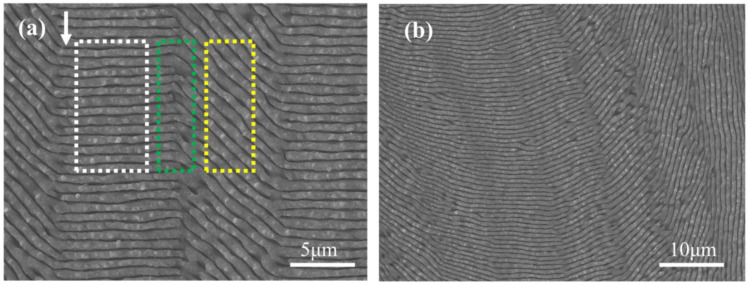
(**a**) SEM image of a double-angle stitched grating. Within the white and yellow rectangular frames are LSFLs generated on a single-crystal silicon (100) surface irradiated by linearly polarized light at polarization angles of 0° and 45°, respectively. The green rectangular frame highlights the area where both sets of LSFLs are stitched together. The direction of the laser scan is indicated by the white single-headed arrow. (**b**) SEM image of a multi-angle stitched grating.

To achieve a large-area meta-surface based on LIPSS arrays with angled stitching on the surface of single-crystal silicon (100), as illustrated in Figure 7, it is crucial to consider the polarization-dependent anisotropy of LIPSSs mentioned above. The white rectangle on the left side of Figure 7a contains LSFLs created at a polarization angle of 0° with a periodicity of 720 nm ± 67 nm and a modification region width of 9.05 μm ± 0.21 μm. Within the yellow rectangle on the right, at the same energy density but with the polarization angle adjusted to 45°, the LSFLs have a periodicity of 780 nm ± 148 nm and a modification region width of 7.45 μm ± 0.22 μm. When stitching these two sets together, in addition to considering the appropriate gap for stitching, it is also necessary to address the drop in stitching quality due to inconsistent periodicity on both sides. For the more complex pattern processing displayed in Figure 7b, resolving the stitching quality issues for LIPSSs at multiple angles is even more complicated. These challenges are not within the scope of this paper; however, in future work, it is hoped that by using different laser wavelengths to affect the periodicity of LIPSSs, it might be possible to compensate for the differences in periodicity between LIPSSs of various polarization states to improve the quality of stitching.

## 4. Conclusions

In this paper, we investigate the formation of LIPSSs on single-crystal silicon surfaces under picosecond laser pulse irradiation with different polarization states relative to crystal orientation and scanning direction. With increasing laser energy density, the LIPSSs’ morphology successively evolves from HSFL_∥_ to LSFL_⊥_ and finally to grooves. Particularly, the coexistence of HSFL_∥_ and LSFL_⊥_ on the same scan line with fixed processing parameters has been observed. Moreover, the study explores the combined effects of laser polarization scanning direction and laser polarization crystal orientation on the generation of LIPSSs on the Si (100) surface. The experimental results indicate that the former affects the overlapping manner of elliptical modified morphologies while the latter influences the LIPSSs generation threshold and results in different modified area sizes at the same laser energy density. The interplay between these two effects impacts the size of the overlapping modified areas and, in conjunction with a feedback mechanism, leads to polarization-dependent anisotropic characteristics in LIPSSs’ morphology, regularity, modification width, and periodicity, which are distinct from those caused by a single effect. When the polarization angle approaches 45°, the regularity of LSFLs deteriorates, the modification width decreases, and the periodicity increases. This is crucial for the precise control of patterned LSFLs processing based on dynamic scanning and polarization control.

## Data Availability

Data are contained within the article.
